# Cytomegalovirus Colitis in Primary Hypogammaglobulinemia With Normal CD4+ T Cells: Deficiency of CMV-Specific CD8+ T Cells

**DOI:** 10.3389/fimmu.2019.00399

**Published:** 2019-03-07

**Authors:** Sudhanshu Agrawal, Amrita Khokhar, Sudhir Gupta

**Affiliations:** Program in Primary Immunodeficiency and Aging, Division of Basic and Clinical Immunology, Jeffrey Modell Diagnostic Center for Primary Immunodeficiencies, University of California, Irvine, Irvine, CA, United States

**Keywords:** PD-1, T effector memory cells, CD4 Treg, CD8 Teg, cytotoxic T cells

## Abstract

CMV colitis has been reported in immunocompromized patients with severe deficiency of CD4+ T cells and T cell functions. In this study we present an extensive immunological analysis in a patient with primary hypogammaglobulinemia and CMV colitis who had normal numbers of CD3+T, CD4+T and CD8+T cells, and normal T cell proliferative responses to mitogens and recall antigens. Naïve (T_N_), central (T_CM_), and effector (T_EM_) memory subsets of CD4+ and CD8+ T cells, Granzyme+ and Perforin+ CD8+ T cells, PD-1+ T cells, CD4 Treg, CD8 Treg, and CMV tetramer specific CD8+ T cells were analyzed with specific antibodies and isotype controls using multicolor flow cytometry. CD8 T_EM_, Granzyme+ and Perforin+, and PD-1 CD8+T cells were increased, whereas CD8 T_N_ and CD8 T_CM_ cells were decreased in the patient as compared to controls. CMV tetramer+ CD8+ T cells were decreased in the patient. These data demonstrate that a deficiency of CMV-specific CD8+ T cells even in the presence of normal CD4+ T cell numbers and normal T cell functions may predispose patients with primary hypogammaglobulinemia to CMV colitis.

## Introduction

Cytomegalovirus (CMV) is a double stranded DNA virus of the herpes virus family, which can lead to a spectrum of clinical presentations, from latent infection to disseminated disease. CMV infection has often been observed in immunocompromized patients with low CD4+ T cell counts, and T cell functions including patients with human immunodeficiency virus infection, solid organ or hematopoietic stem cell transplant recipients, and those on immunosuppressive agents (1–5). Rare gastrointestinal infections with CMV have been described in patients with common variable immunodeficiency (6–15). However, these patients had low CD4+ T cells, and in none of these studies detailed immunological analyses, including CMV-specific CD8+ T cells were reported. We present, to best of our knowledge, the first detailed immunological analysis in a patient with primary hypogammaglobulinemia and CMV colitis, who has normal numbers of CD3+, CD4+, and CD8+ T cell subsets, and normal responses to mitogens and soluble antigens. Patient displayed a deficiency of CMV-specific CD8+ T cells, and expansion of PD-1+ exhausted CD8+ T cells.

## Background

### Case

A 39-year-old male with a past medical history significant for hypogammaglobulinemia, and asthma, and colectomy status-post bowel perforation, presented with several days of increasing watery ostomy output, non-bloody vomiting, and subjective fevers. The cause of spontaneous bowel perforation is unclear. The diagnosis of hypogammaglobulinemia had been made 1 year prior to presentation when patient had no prior history of any gastrointestinal symptoms. Therefore, excluding any possibility of hypogammaglobulinemia secondary to protein-losing enteropathy. He had been doing well on intravenous immunoglobulin (IVIG) up until this point. A computed tomography scan of the abdomen and pelvis with contrast revealed diffuse small bowel mucosal hyperenhancement consistent with enteritis, with no evidence of free air or recurrent bowel perforation. He underwent EGD and colonoscopy with no complications. Both procedures revealed grossly normal mucosa with the exception of two diminutive sessile polyps at the cecum, which were biopsied. Immunohistochemistry revealed cells positive for cytomegalovirus and evidence of chronic active crypt-destructive colitis related to cytomegalovirus infection. Serum CMV was quantitated by PCR and found to be 9561 IU/ml. He was subsequently started on valgancyclovir with marked improvement in his clinical condition. Results of routine immunological analysis prior to starting immunoglobuline therapy revealed IgG (498 mg/dl; control range 694–1,618 mg/dl), IgA (118 mg/dl; control range 68–378 mg/dl), IgM (92 mg/dl; control range 65–263 mg/dl). At the time of diagnosis of CMV colitis, his lymphocyte subsets were as follow: CD3+ T cells 1,828/μl (control range 502–1,902/μl), CD4+ T-cells 949/μl (control range 338–1,194/μl), CD8+ T-cells 970/μl, (control range 85–729/μl), CD19+ B-cells 86/μl (control range 51–473/μl), and NK cells 86/μl (range 12–349/μl). Proliferative responses to recall antigens (*Candida albicans* and tetanus toxoid) and mitogens (phytohemagglutinin, concanavalin A, and pokeweed) were also normal. HIV was negative.

## Materials and Methods

### Subjects

Peripheral blood mononuclear cells (PBMCs) were isolated from blood of patient and healthy subject by Ficoll-hypaque density gradient. Healthy controls were age-matched CMV antibody positive males. The protocol was approved by the Human Subject Committee of the Institution Review Board of the University of California, Irvine. A written informed consent has been obtained from the patient for the publication of this case report, including any accompanying images or data contained within the manuscript.

#### Antibodies and Reagents

CD4 PerCP, CD8 PerCP, CD45RA APC, CCR7FITC, CD183 PE, Foxp3 PE, CD170a PE, Granzyme-B FITC, Perforin FITC, and PD-1 APC antibodies were purchased from BD Parmingen (San Jose, California). iTAg MHC tetramer HLA-A^*^0201 and CMV PP65 Tetramer PE were obtained from MBL International corps (Woburn MA).

#### Immunophenotype of Subsets of CD4+ and CD8+ T Cells

PBMNCs Cells were incubated with various monoclonal antibodies and isotype controls for 30 min at room temperature in dark, washed, and acquired by FACSCalibur and analyzed using Flowjo software (Treestar, Ashland, Oregon). Subsets of CD4+ and CD8+ T cells were identified as naïve (T_N_): CCR7+CD45RA+, central memory (T_CM_): CCR7+CD45RA-, effector memory (T_EM_): CCR7-CD45RA-, and terminally differentiated effector memory (T_EMRA_): CCR7-CD45RA+), and exhausted PD-1+ CD8+ T cells.

#### Cytotoxic CD8+ T cells

PBMCs were activated with anti-CD3/CD28 for 24 h, and then stained with CD8PerCP and CD107a PE for surface staining for 30 min. Cells were then fixed and permeabilize by fix perm buffer (BD biosciences), and stained with Granzyme B-FITC and Perforin-FITC, respectively and appropriate isotypes.

#### CMV Tetramer Staining

PBMCs were activated with anti-CD3/CD28, and samples were collected at day 1 and day 4. Cells were stained with CD8 PerCP and HLA-A^*^0201 CMV PP65 Tetramer PE. After staining the cells were washed with PBS and analyzed by FACSCalibur (BD Biosciences, San Jose, CA) equipped with argon ion laser emitting at 488 nm (for FITC, PE and PerCP excitation) and a spatially separate diode laser emitting at 631 nm (for APC excitation). Forward and side scatters were used to gate and exclude cellular debris. Ten thousand cells were acquired and analyzed using Flowjo software.

#### CD4 and CD8 T Regulatory Cells

For CD4 Treg, cells were stained with CD4PerCP, CD25 FITC, and CD127 Alexa647, and for CD8 Treg, cells were stained with CD45RA APC, CCR7FITC, CD183 PE, according to manufacturer's protocol, followed by Foxp3 intracellular staining with Foxp3 PE monoclonal antibody and an appropriate isotype control (Mouse IgG 1k-PE) were used to evaluate nonspecific staining and set using a Human Foxp3 Buffer Set. Staining procedures was performed according to the manufacturer's recommendation. In the population of CD4+ T cells, Treg cells were identified as CD25^high^CD127^Low^Foxp3+ cells, and in CD8 T cells Treg were identified as CD183+CCR7+CD45RA-FoxP3+ Cells, and acquired with FACSCalibur and analyzed by Flowjo software.

## Results

### Altered Naïve and Memory Subsets of CD4+ and CD8+ T Cells

**Figure 1:**
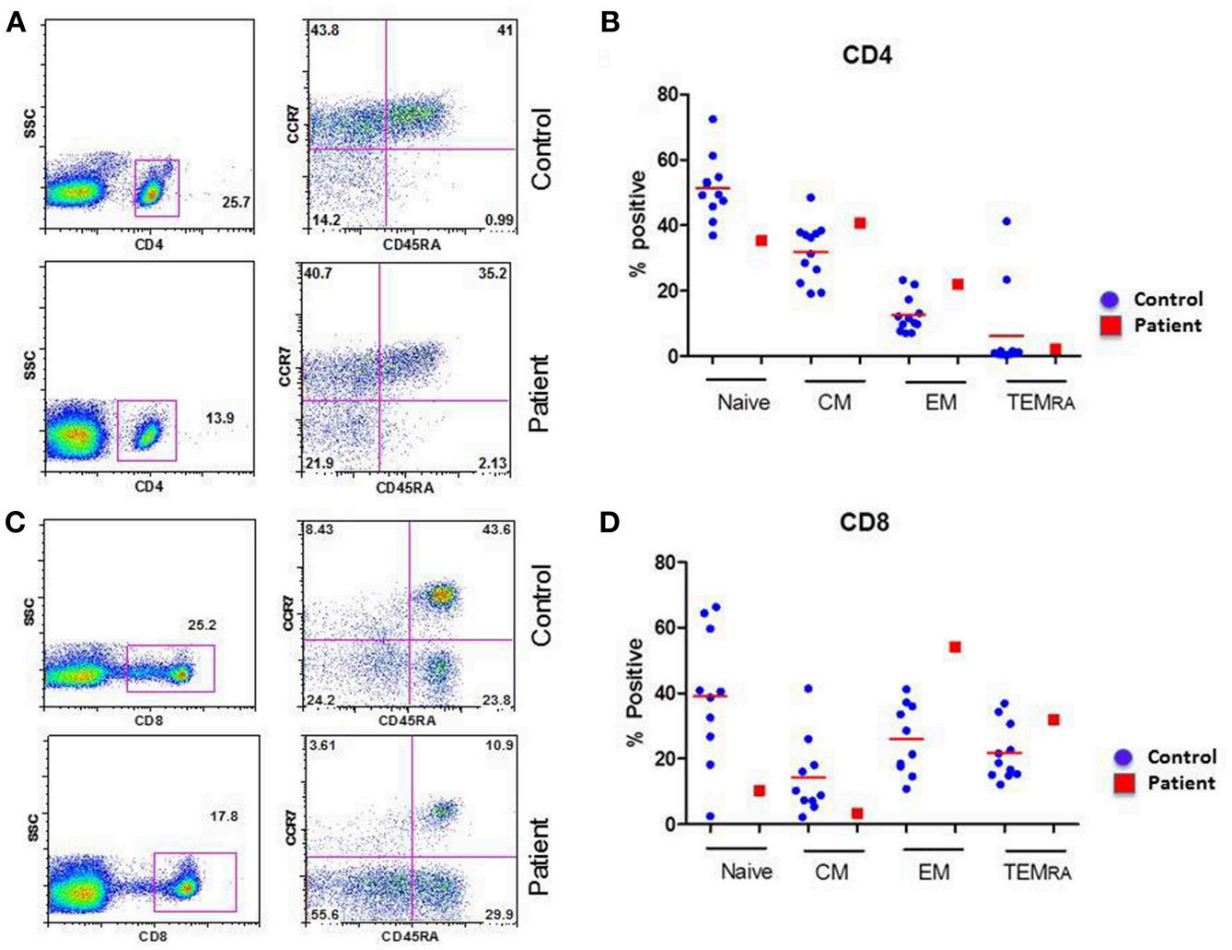
Subsets of CD4+ **(A,B)** and CD8+ **(C,D)** T cells. **(A,C)** are flow cytograph from the patient and a simultaneously analyzed healthy control. **(B,D)** show individual data from 10 healthy controls and the patient. CD8 T_EM_ cells are increased, and CD8 T_N_ and CD8 T_CM_ cells are decreased in the patient.

CD4+ and CD8+ T cells, based upon their homing patterns, phenotypic expression of chemokine receptors, and effector functions have been subdivided into naïve (T_N_), central memory (T_CM_), effector memory (T_EM_), and terminally differentiated effector memory (T_EMRA_) subsets (16–18). Therefore, we examined these subsets in the patient and controls. A flow cytograph of patient and simultaneously studied control for CD4+ T cell subset is shown in Figure 1A, and for CD8+ T cells in Figure 1C. Individual data from 10 healthy normal control and compared with the patient for CD4+ and CD8+ T cells subsets, respectively are shown in Figures 1B,D. CD8+CCR7-CD45RA**-**T_EM_ were increased, whereas CD8+CCR7+CD45RA+T_N_ and CD8+CCD7+CD45RA- T_CM_ cells were decreased in the patient as compared to controls.

### CD4+ T Regulatory and CD8+ T Regulatory Cells Are Decreased

**Figure 2:**
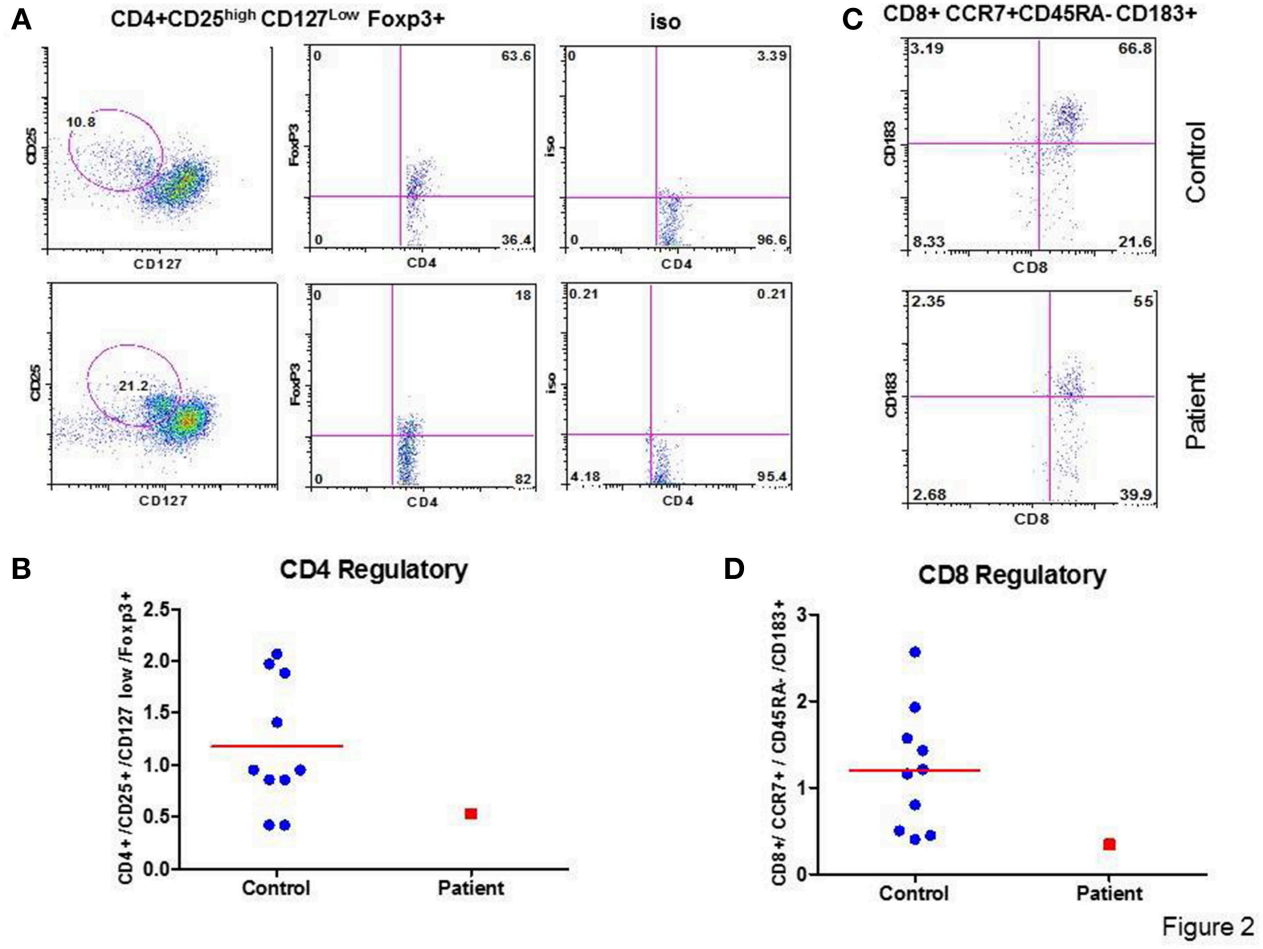
CD4 Treg and CD8 Treg. Flow cytograph for CD4 Treg **(A)** and CD8Treg **(C)** in the patient and a simultaneously-analyzed healthy control. Individual data from 10 healthy controls and the patient for CD4 Treg **(B)** and CD8 Treg **(D)** are shown. CD4+ Treg and CD8+ Treg are decreased in the patient.

A role of CD+ Treg cells in regulating immune response is well-established (19); however, a role of CD8+ Treg is emerging (20). Therefore, we analyzed the proportions of CD4+ T cells and CD8+ Treg in the patient and control. In the patient, proportion of CD4+CD25^high^CD127^low^FoxP3+ Treg and CD8+CCR7+CD45RA-CD183+FoxP3+Treg were decreased as compared to CD4 Treg and CD8 Treg to healthy controls. Figures 2A,C show flow cytographs of CD4+ Treg and CD8+ Treg respectively, for the patient and control studied simultaneously; Figures 2B,D show individual data for CD4 Treg and CD8 Treg from 10 healthy controls and the patient.

### Perforin and Granzyme B Positive CD8+ T Cells Are Increased

**Figure 3:**
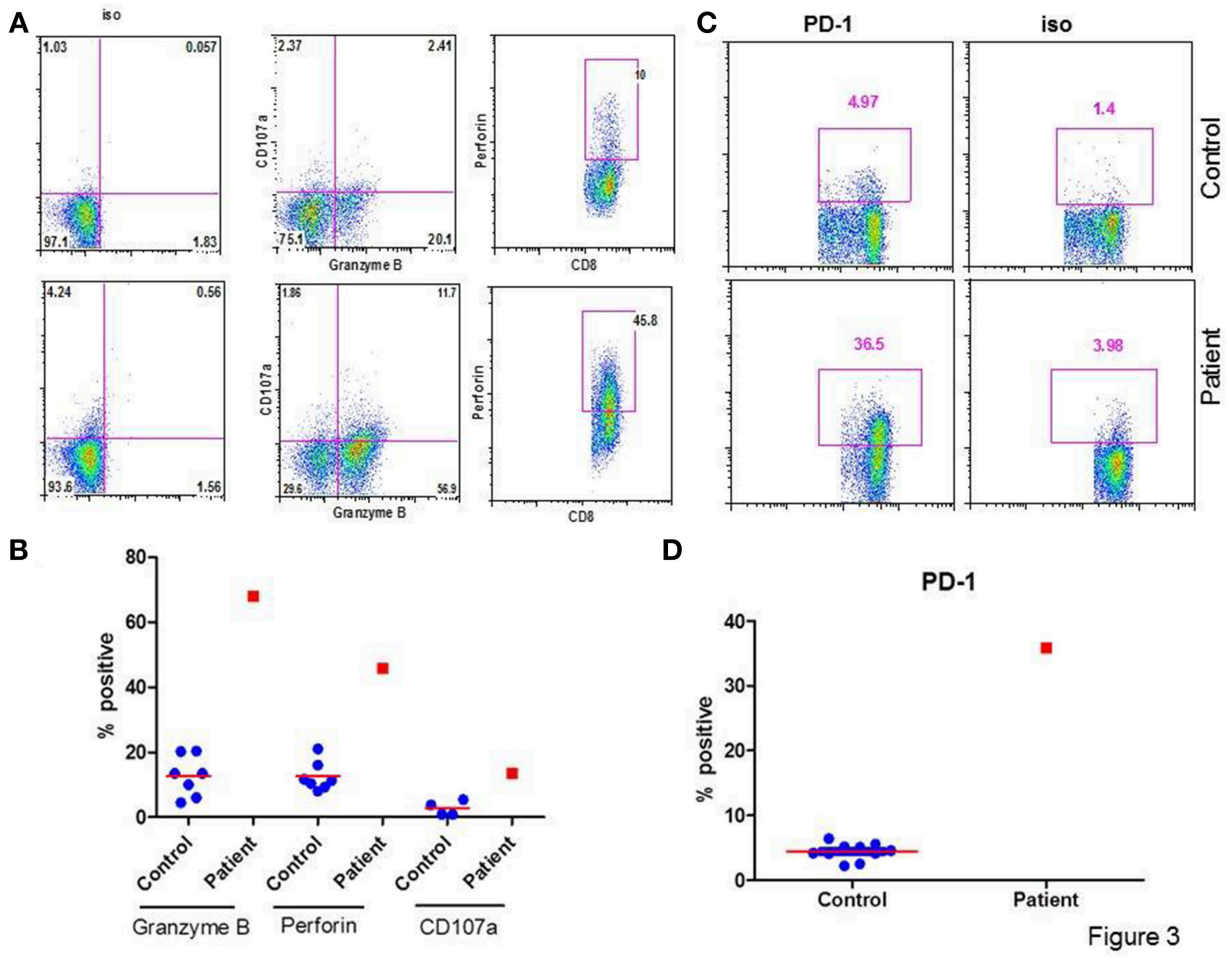
Granzyme B+, Perforin+, and PD-1+ CD8+ T cells. **(A)** shows a flow cytograph for 107a+, granzyme B+, and perforin + CD8+ T cells. **(B)** shows individual data from 5 healthy control subjects and the patient. **(C)** shows a flow cytograph of PD-1+CD8+ T cells in the patient and a simultaneously-analyzed healthy control. **(D)** show individual data of PD-1+ CD8+ T cells in 10 healthy controls and the patient. Granzyme B+, CD107a+, Perforin +, and PD-1+ CD8+ T cells are increased in the patient.

Figure 3A show a flow cytograph from the patient and a simultaneously studied healthy control, and Figure 3B show individual data from 10 healthy controls and the patient. The proportion of cytotoxic (107a+ Granzyme B +) and perforin+ CD8+ T cells were increased in the patient as compared to controls. Figure 3C show a flow cytograph of PD-1+ CD8+ T cells from the patient and simultaneously studied healthy control, and Figure 3D show individual data from 10 healthy controls and the patient. PD-1+CD8+ T cells were markedly increased as compared to healthy control.

### CMV-Specific CD8+ T Cells Are Decreased

**Figure 4:**
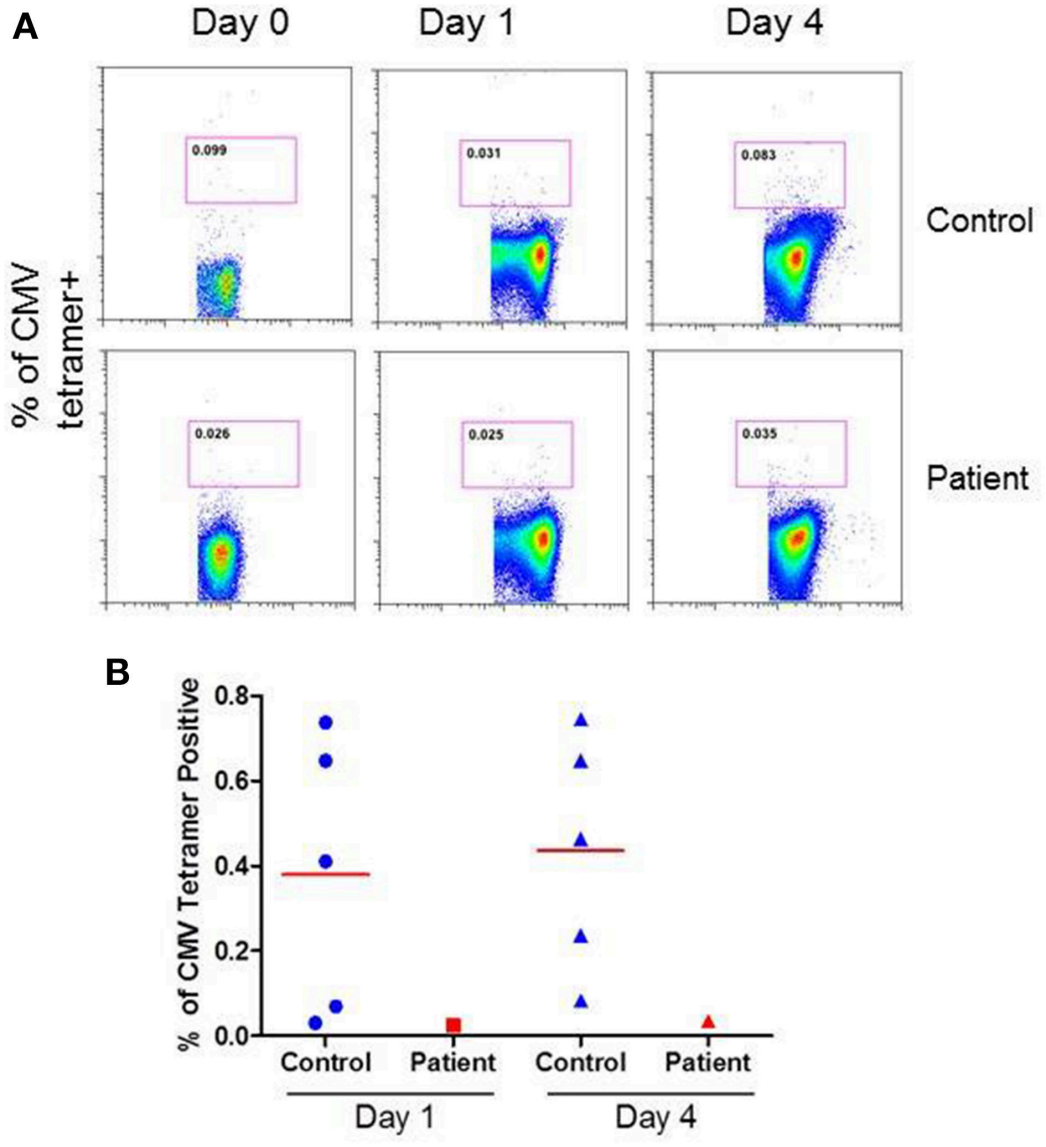
CMV-specific tetramer+ CD8+ T cells at day 1 and 4 following activation with anti-CD3/CD28 antibody. **(A)** show a flow cytograph of the patient and simultaneously studied healthy control, and **(B)** shows individual data from 5 healthy controls and the patient. CMV-specific tetramer + CD8+ T cells are decreased in the patient.

CMV-specific tetramer + CD8 T cells were analyzed at both day 1 and day 4 following activation with anti-CD3/CD28 antibody. Figure 4A show a flow cytograph from the patient and a simultaneously studied healthy control. At day 4, <50% of CMV tetramer positive cells (0.035%) were present in the patient as compared to healthy control (0.083%). A repeat CMV-specific tetramer+ CD8 T cells test 4 weeks later revealed similar results (data not shown). Figure 4B shows individual data from 5 healthy subjects and the patient. The patient had decreased CMV-specific CD8+ T cells.

## Discussion

The cytotoxic T lymphocytes (CTLs) play an important role in defense against infection with CMV, and CMV tetramer-specific CTLs are routinely found in healthy seropositive patients (21). CMV enteritis and colitis are frequently observed in immunocompromized patients with severe depletion of CD4+ T cells (6–15). CMV colitis has also been described in immunocompetent patients (124–25). However, these patients may not be immunocompetent. Majority of these patients had comorbidities that may be associated with immune dysfunctions including diabetes mellitus, cirrhosis of liver, end-stage renal disease, respiratory failure for which they were admitted to ICU, and untreated non-hematological malignancies. No immunological data were reported in these studies. CMV infection of gastrointestinal tract in primary antibody deficiency diseases is infrequent. The majority of reported cases of CMV colitis in primary antibody deficiency disorders have been limited to patients with common variable immunodeficiency (CVID) (6–15). However, in these patients either CD4+ T cell numbers were low or not reported. In none of these studies detailed immunological analyses were performed. In immunosuppressed patients who are susceptible to CMV reactivation the specific to CMV epitope pp65 CD8 repertoire is limited. However, our patient has no evidence of immunosuppression. Our patient had normal number of CD3+T, CD4+T, NK, B cells, and CD8+T cells were increased. He also responded normally to mitogens and recall antigens, suggesting normal T cell functions. Our patient has CMV-specific CD8+ T cell functional defect (lacunar immunodeficiency).

T cells have been further classified into naïve (T_N_), central memory (T_CM_), effector memory (T_EM_), and terminally differentiated effector memory (T_EMRA_), and have been characterized extensively for phenotype and functions (16–18, 22). Naïve T cells (T_N_) upon exposure to virus undergo a clonal expansion of effector cells, which after clearance of viral antigens undergo a phase of contraction when antigen-specific T cells undergo apoptosis, and a small number of antigen-specific T cells are retained as memory T cells. These memory T cells differentially express adhesion molecules and chemokine receptors, which allow them to migrate and home in peripheral blood lymphoid (central memory, T_CM_), or extralymphoid tissues (effector memory, T_EM_). A small subpopulation of T_EM_ cells that re-acquires CD45RA are termed as T_EMRA_ or terminally differentiated memory cells. T_EM_ and T_EMRA_ T cells display poor proliferation, decreased telomere length, resistance to apoptosis, and express greater granzyme and perforin as compared to T_N_ and T_CM_. Perforin, a cytolytic protein, and granzyme B (GrB), a serine protease, are found in granules in cytotoxic T-cells and natural killer cells. Together they help mediate apoptosis in targeted cells, including virus-infected target cells (23). In our patient, CD8+ T_EM_ cells are increased which would be consistent with increased granzyme B+, and perforin+ CD8+ T cells.

Programmed cell death protein 1, or PD-1, functions as an immune checkpoint and is found on exhausted T-cells (24). Sester et al. (25) showed that in a population of renal transplant recipients, CMV replication was associated with higher expression of PD-1 in CMV-specific T cells. Dirks et al. (26) observed that high PD-1 expressing CMV-specific T cells proliferate poorly. Our patient also has increased PD-1+ CD8+ T cells. This would suggest that CD8+ CTL have expanded in response to CMV infection; however, in doing so they appear to be exhausted (PD-1+), and therefore, resulting in the persistence of CMV.

Lamba et al. (27) studied the incidence and recurrence of CMV infection in the patients who received reduced intensity conditioning for allogeneic stem cell transplantation. Those patients who failed to generate a CMV-specific CTL response went on to develop CMV infection; one of these patients developed CMV colitis. Cummins et al. (28) also found decreased or absent CMV-specific T cells in two patients with late onset CMV disease after organ transplantation; both of these patients also presented with colitis. In our patient, CMV-specific CD8+ T cells were also reduced. Therefore, CMV-specific CD8+ T cell deficiency may predispose patients with antibody deficiency with normal T cell and T cell subsets, and NK cells to the development of CMV colitis.

CD4+ T regulatory cells (Treg) suppress the activity of CMV-pp65-specific CD8+ T cells, as measured by IFN-γ production (29). In our patient CD4+ Treg were decreased. Therefore, alteration in CD4 Treg in our patient is unlikely to be responsible for decreased CMV-specific CD8+ T cells. We also observed decreased CD8+ Treg in our patient. A role of CD8+ Treg in regulating CMV-specific effector response, however, has not been explored.

## Concluding Remarks

A deficiency of CMV-specific cytotoxic CD8+ T cells may predispose patients with antibody deficiency and normal numbers of CD4+ T cells and functions to CMV colitis. Furthermore, an expansion of PD-1+ exhausted CD8+ T cells may play a role in the persistence of CMV infection. The mechanism (s) for deficiency of CMV-specific CD8+ T cells remains unclear. Therefore, patients with antibody deficiency and CMV c CMV-specific CD8+ T cells.

## Ethics Statement

This study was carried out in accordance with the recommendations outlined in the Belmont Report, and UCI will apply DHHS regulations (45 CFR 46, including all Subparts) to all federally-funded proposed research involving human participants. Commensurate protections are in place for all other human subject research conducted at or under the jurisdiction of UCI. UCI agrees to apply additional regulations such as the U.S. Food and Drug Administration Human Subject Regulations (21 CFR 50, 56, 312, and 812) and the Health Insurance Portability and Accountability Act of 1996 (HIPAA), when applicable, to research involving human participants. The protocol was approved by the IRB committee (Human) of the University of California, Irvine. All subjects gave written informed consent in accordance with the Declaration of Helsinki.

## Author Contributions

SA performed all flow cytometric analysis. AK collected clinical data and wrote initial draft. SG initiated the work-up of the case, guided both SA and AK and expanded and finalized the manuscript.

### Conflict of Interest Statement

The authors declare that the research was conducted in the absence of any commercial or financial relationships that could be construed as a potential conflict of interest.
